# At the Interface of Lifestyle, Behavior, and Circadian Rhythms: Metabolic Implications

**DOI:** 10.3389/fnut.2019.00132

**Published:** 2019-08-28

**Authors:** Seul-A Bae, Ming Zhu Fang, Vinod Rustgi, Helmut Zarbl, Ioannis P. Androulakis

**Affiliations:** ^1^Chemical and Biochemical Engineering Department, Rutgers University, Piscataway, NJ, United States; ^2^Department of Environmental and Occupational Medicine, Robert Wood Johnson Medical School, Piscataway, NJ, United States; ^3^National Institute for Environmental Health Sciences (NIEHS) Center for Environmental Exposures and Disease, Environmental and Occupational Health Sciences Institute, Piscataway, NJ, United States; ^4^Cancer Institute of New Jersey, Rutgers, The State University of New Jersey, Piscataway, NJ, United States; ^5^Division of Gastroenterology and Hepatology, Department of Internal Medicine, Rutgers Robert Wood Johnson Medical School, New Brunswick, NJ, United States; ^6^Biomedical Engineering Department, Rutgers University, Piscataway, NJ, United States; ^7^Department of Surgery, Rutgers Robert Wood Johnson Medical School, New Brunswick, NJ, United States

**Keywords:** metabolic syndrome, circadian rhythms, chronodisruption, shift work, metabolism

## Abstract

Nutrient metabolism is under circadian regulation. Disruption of circadian rhythms by lifestyle and behavioral choices such as work schedules, eating patterns, and social jetlag, seriously impacts metabolic homeostasis. Metabolic dysfunction due to chronic misalignment of an organism's endogenous rhythms is detrimental to health, increasing the risk of obesity, metabolic and cardiovascular disease, diabetes, and cancer. In this paper, we review literature on recent findings on the mechanisms that communicate metabolic signals to circadian clocks and *vice versa*, and how human behavioral changes imposed by societal and occupational demands affect the physiological networks integrating peripheral clocks and metabolism. Finally, we discuss factors possibly contributing to inter-individual variability in response to circadian changes in the context of metabolic (dys)function.

## Introduction

Organisms have evolved to anticipate and adapt to the cyclic nature of the environment on earth, most notably diurnal variations in sunlight, and temperature over the earth's 24-h rotation about its axis. The circadian timing system aligns oscillations in biological processes such as food intake, alertness, and energy expenditure to the earth's solar day, producing rhythms in physiology, and behavior in organisms ranging from bacteria to mammals (1). The alternating environmental cues such as the light/dark cycle, changes in temperature, and availability of food, act as *zeitgebers (timekeepers)*, that synchronize the endogenous timing systems of every cell in an organism. A variety of physiological activities, including immune response, metabolism, sleep cycles and endocrine signaling, are under circadian regulation and modulation. Of importance is the reciprocal control between the circadian clock and metabolism, whose sensitivity to a variety of genetic and behavioral factors leads to disruption of the rhythms and consequently, metabolic dysfunction.

The endogenous time keeping system is organized as a hierarchical, interconnected network of clocks. The clocks receive input from environmental cues external to the host, processes them to generate a rhythm, which then regulates various output pathways which, in turn, regulate key biological functions (2). The central clock lies in the suprachiasmatic nucleus (SCN) of the brain and oscillates in an autonomous manner (3). The central clock synchronizes the network of peripheral clocks, which are present in all tissues and cells (4). The synchronization of peripheral clocks is necessary for proper metabolic, physiological, and behavioral processes of the host. The central pacemaker is synchronized to the cycle of the ambient light that in mammals is exclusively detected by specialized receptor cells the eyes. Rods, cones, and melanopsin containing retinal ganglion cells process the light and transduce signals to the SCN via neuronal pathways (5, 6). However, the light/dark cycle is not the only *zeitgeber*. Circadian clocks are also under the control of energy intake and the timing thereof, ensuring that the peripheral rhythms can be altered to the food availability, effectively misaligning the rhythms in the periphery from the phase in the SCN. In return, circadian clocks regulate components of metabolism by controlling the rhythmic expression of genes encoding regulators and enzymes in various metabolic pathways. Feeding rhythms influence key clock components and more importantly clock outputs via enzymatic reactions and transcriptional regulations (7–9).

The intricate relationship between circadian rhythms and metabolism of glucose, lipid, amino acids, etc. has been studied in the context of enzymes and hormones involved in digesting the nutrients and their interaction with peripheral clocks (10, 11). The rest/active cycles and fast/feed cycles that mammals experience diurnally lead to alternating nutrient supply throughout the day. The metabolic organs of the host must manage these diurnal fluctuations in nutrients while also maintaining physiological homeostasis (12). The circadian regulation of metabolism is thought to provide the host organism with the flexibility in regulating metabolic activities in response to changing environmental conditions. Considering the intricate relationship between circadian rhythms and metabolism, it is not surprising that chronic disruption of circadian rhythms is associated with the development of metabolic syndrome, which may lead to cardiovascular diseases and diabetes (13–17). Furthermore, time-restricted feeding studies (restricted temporal access to food without calorie restriction) in mice has shown that metabolic cues influence circadian rhythmicity enabling, for instance, restoring circadian oscillations of some peripheral clock components in clock-deficient mouse livers (18).

In this paper, we review literature on the networks of metabolic signals and circadian clocks, and how human behaviors driven by societal demands and lifestyle choices affect the physiological networks integrating peripheral clocks and metabolism. Understanding of these networks can facilitate future research in overcoming metabolic syndrome due to chronodisruption by pharmacological means and behavioral modification. We will first discuss the molecular basis on the interaction between clock and metabolic components. We discuss the extended network of interactions between endogenous rhythms and the entraining environmental cues. Then we will review a few behaviors (for both human and animals) that are known to induce metabolic syndrome associated with chronodisruption. We discuss shift work, irregular meals, and social jetlag as behaviors which cause misalignment between the peripheral and central clocks. We further elaborate on sex differences, microbiota, genetic makeup, including race and ethnicity, and lifestyle as the factors that generate interindividual variability in circadian rhythms manifesting to metabolic activities, as these factors may prove useful in developing personalized care for patients of metabolic syndrome. Finally, we provide a brief overview of mathematical models that aim to understand the bi-directional regulation that occurs between peripheral clocks and metabolism, which aim to develop pharmacologic approaches targeting the circadian clock in the context of metabolic disorders.

## The Mammalian Circadian System

In mammals, the circadian clock system is organized into a distributed, hierarchical system composed of the central pacemaker that resides in the SCN in the brain and peripheral oscillators synchronized to the central clock (19). The clocks give rise to rhythms in hormonal and metabolic signaling even in the absence of environmental cues, and the signaling establishes phase relations among the various peripheral clocks. These rhythmic signals play a major in regulating immune and metabolic functions (20, 21). The ability for mammalian organisms to diverse biological functions in a temporal manner confers and adaptive advantage as they cope with 24-h changes in the environment. The central clock in the SCN receives the photic input through the retinal ganglion cells in the eye (5, 6), and transduces the information to the peripheral clocks, within other brain regions, and outside of the brain. The necessity of proper SCN function is evident in studies with SCN lesioned animals and tissue explants (22, 23), where the phase synchrony among the cells were gradually decreased over time due to period differences in individual cells. The peripheral clocks then drive tissue specific processes. It is now well-recognized that in order to benefit the host, the local, and central rhythms must be synchronized to each other as well as to the outside environment. The oscillators have identical molecular makeup in the SCN neurons and the peripheral cells, with the peripheral oscillators coupled to the SCN via synaptic and paracrine signals (24).

The autonomous oscillation of the circadian clocks is driven transcriptional and translational feedback loops of clock genes. The CLOCK and BMAL1 form a heterocomplex and activate transcription of their own repressors, *Per* and *Cry* (25). CLOCK/BMAL1 heterodimers bind to the E box region of *Per1/2* and *Cry 1/2* genes and activate the transcription of these genes. PER and CRY proteins accumulate as a result of this transcriptional activation and form the PER/CRY heterodimer complexes, which translocates to the nucleus (26). The auto-repression of *Per* and *Cry* genes by their own products, PER and CRY, occurs through inhibition of CLOCK/BMAL1 induced transcription by the nuclear PER/CRY complex (25). The PER/CRY complex also stimulates the expression of BMAL1 (27–29). Aside from PER and CRY, the CLOCK/BMAL1 heterocomplex also transcriptionally activates the nuclear receptors REV-ERBs and RORs (25). REV-ERBs represses the transcription of *Bmal1* while RORs activate it (25, 30).

## Circadian Clock and Metabolism Crosstalk

In this section we discuss the molecular basis for the interaction between circadian clock and metabolism. In particular, we discuss the role or nutrient sensors that bridge the circadian clocks to energy metabolism, since metabolic syndrome linked with behavior-induced circadian disruption often involve taking in nutrients at naturally inactive times. The nutrient sensors can entrain the local clocks in the metabolic tissues away from the SCN due to the nutrient cues, causing a phase misalignment between the central and peripheral clocks and leading to a break in metabolic homeostasis. Nutrient sensors such as sirtuins (SIRTs), AMP-activated protein kinase (AMPK), and poly ADP-ribose polymerase 1 (PARP1) exhibit circadian behavior and interact with core components of the clock system, while also playing key roles in metabolic activities (31, 32).

SIRTs are NAD^+^ activated class III histone deacetylases (HDAC), homologous to Sir2 (silence information regulator 2) in yeast (8). Among the 7 members of mammalian sirtuins, SIRT1, SIRT3, and SIRT6 interact with circadian genes. SIRT3 is involved in the circadian mitochondrial function (33), while SIRT6 controls the circadian clock at a chromatin level (34). SIRT6 is involved in the recruitment of the circadian transcriptional machinery (CLOCK and BMAL1) to E-box containing core clock gene promoters (35). According to microarray analysis of hepatic sirt1 and sirt6 deficient mice, SIRT6 regulates a set of core clock output genes different from those regulated by SIRT1 (34).

The CLOCK/BMAL1 complex also acts with SIRT1 to transcriptionally activate nicotinamide phosphoribosyltransferase (NAMTP) the rate-limiting enzyme of the NAD^+^ salvage pathway (36). SIRT1 is a key candidate for bridging the circadian clocks and metabolism. The activity of SIRT1 occurs in the nucleus, modulating metabolism of lipids, proteins, and carbohydrates, and enhancing mitochondrial activity (37). As such, SIRT1 has been studied extensively in the context of oncogenesis, aging, and metabolism (38). SIRT1 acts as a nutrient sensor as its enzymatic activity requires binding of nicotinamide adenine dinucleotide (NAD^+^) into its catalytic site along with the substrate. Understanding the activity of SIRT1 would require an accurate portrayal of the NAD^+^ level in the cell, since it directly activates SIRT1. In addition to synthesizing NAD^+^ from amino acids, cells can also recover NAD^+^ from the NAD^+^ salvage pathway (39), where nicotinamide (NAM) is released from NAD^+^, NAM is converted to nicotinamide mononucleotide (NMN), and NMN is converted back to NAD^+^, completing the cycle.

NAD^+^, the oxidized form of the nicotinamide adenine dinucleotide, serves an important role in linking clocks to metabolism, acting as the cellular energy sensor and modulating the activity level of SIRT1. In liver, NAD^+^ level oscillates throughout the 24-h day (40), due to contributions from a variety of factors including the feeding/fasting state of the host, oscillations in the NAMPT activity, and activation of PARP-1 (7). SIRT1 is co-activated by NAD^+^ through direct binding, and regulates the transcriptional activity of CLOCK/BMAL1, creating a bi-directional relationship between the circadian clocks and metabolism (36). Additionally, SIRT1 mediates deacetylation and thereby degradation of PER2 protein (41). SIRT1 activity has complex relationships with the NAD^+^ salvage pathway and the peripheral clocks. First, SIRT1 activity requires binding of NAD^+^, thus NAD^+^ has an activating effect on SIRT1. Second, SIRT1 activity is inhibited by NAM, the precursor of the rate-limiting step. Third, expression of nicotinamide mononucleotide adenylyltransferase (NAMPT), the rate-limiting enzyme of the NAD^+^ salvage cycle, is under the control of SIRT1, which is a co-transcription factor for the NAMPT gene along with peripheral clock genes (PCGs). In summary, SIRT1 functions as a nutrient sensor, being under the influence of the energy state of the cells, represented by NAD^+^. It is also under the effect of the circadian rhythmicity presented by NAD^+^, NAM, and NAMPT. Another potential NAD^+^ sensor is PARP-1, whose activity is also connected to circadian gene expression. When mice transition from *ad libitum* feeding to day-time feeding, the phase inversion for clock components occurs more slowly in *Parp-1* knockout animals, indicating that PARP-1 is involve in entraining the circadian rhythms in the periphery (42).

Like the NAD^+^ levels, the AMP/ATP ratio depends on the feeding/fasting state of the host. AMP-dependent protein kinase (AMPK) responds to the AMP/ATP ratio; when the ratio increases, AMP binds to the γ subunit of AMPK and allosterically modifies the α subunit to be phosphorylated (43). Active AMPK influences the circadian clock by directly phosphorylating and facilitating the degradation of CRY protein (44). Thus, AMPK activity level oscillates with a 24-h period, antiphasic to CRY protein oscillation. AMPK also appears to promote the degradation of PER2 protein indirectly (45).

Peroxisome proliferator-activated receptors (PPARs) are a group of nuclear receptors that function as transcription factors that play a vital role in energy metabolism (46), and also serve as a linker between circadian clocks and metabolism. Recent advances in understandings of PPARs show that all three isoforms of PPARs (α, β/δ, and γ) are expressed and function in rhythmic manner (47). PPARα and PPARγ regulate clock components *Bmal1* and Rev-erbα (48–50). PPARα is also a target of BMAL1 and CLOCK (51).

**Figure 1:**
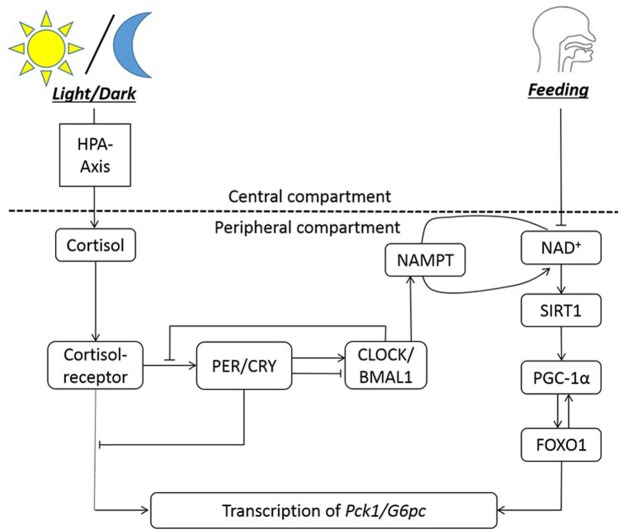
The circadian regulation of hepatic gluconeogenesis illustrates an example of bi-directional regulation between clock genes and metabolism. The HPA axis is entrained to the light/dark cycle, and NAD^+^ availability is entrained to feeding rhythms. NAMPT facilitates NAD^+^ salvage pathway. NAD^+^ co-activates SIRT1, which then activates PGC-1α. PGC-1α activated FOXO1 is necessary for transcription of Pck1 and G6pc genes, along with the cortisol-receptor complex. Cortisol bound to its receptor entrains the clock genes to the light/dark cycle. In summary, the transcription of gluconeogenic genes (Pck1/G6pc) is regulated by cortisol-receptor complex, clocks, and FOXO1.

The nutrient sensors that interact with peripheral clocks bridges the clocks to metabolism of nutrients by regulating the expression of metabolic enzymes and hormones. We illustrate this with clock and metabolic components involved in gluconeogenic gene expression, shown in Figure 1. The fasting/feeding state of the host changes the NAD^+^ level, eventually influencing the transcription of gluconeogenic genes such as *G6pc* and *Pck1* to control the generation of glucose (52). From the *G6pc* genes, G6PC (Glucose-6-phosphatase, catalytic subunit) is expressed, which hydrolyzes glucose-6-phosphate into glucose. The *Pck1* gene encodes for PEPCK (Phosphoenolpyruvate carboxykinase), which is the catalyzer for conversion of oxaloacetate into phosphoenolpyruvate and carbon dioxide. These two enzymes are involved in the very beginning and end of the chemical steps for endogenous production of glucose in liver. When the host experiences long-term fasting (more than 6 h), SIRT1 upregulates the transcription of the two genes encoding for these enzymes. One of the mechanisms in which the transcription of *G6pc* and *Pck1* is initiated is through SIRT1-mediated deacetylation and activation of PPARγ-coactivator α (PGC-1α) and Forkhead box O1 (FOXO1) (53), with the aid of hepatic nuclear factor-4α (HNF-4α) (54). Among a series of rodent studies, a restricted feeding study demonstrates the alteration in G6Pase and PEPCK level and activity due to circadian disruption. The activity of the two enzymes peak slightly before the dark phase in rats fed *ad libitum*, in 12:12-h light/dark cycle. When rats are fed for restricted period during the light phase, G6Pase activity peaks right before the feeding time. T6Pase protein amount oscillate in a bimodal way, with the major peak slightly before the feeding time. For PEPCK, enzyme activity oscillates bimodally with the major peak at the transition from dark to light phase, and protein amount peaks around 2 h after the feeding start time (55).

NAD^+^ is also consumed as a substrate of SIRT3, a mitochondrial Sirtuin protein that also functioning as a protein deacetylase (56). Circadian regulation of *Nampt* transcription by CLOCK, BMAL1, and SIRT1 governs the amount of NAD+ and regulates the deacetylation activity of SIRT3 (56), which activates several key enzymes involved in the citric acid cycle (57) and fatty acid metabolism (58, 59) in mitochondria. Therefore, circadian regulation of mitochondrial function is another pathway to control metabolism by circadian clock (60).

Recent findings suggest that mTOR signaling pathway has interactions with the circadian clock. mTOR signaling centers on mTORC1 and mTORC2, which are multiprotein complexes that involves mTOR, a serine/threonine protein kinase (61, 62). Activated mTOR signaling regulates some of the fundamental biological process including lipid and glucose metabolism (61, 62). One of the ways in which mTOR controls the circadian clocks is through the regulation of photic entrainment of the SCN via mTORC1 (63). mTORC1 is involved in the synchronization of SCN neurons (64). In the periphery, mTOR integrates intracellular signals that involve energy status and nutrients, and can therefore serve to link the cellular metabolic state and circadian clocks, as revealed by RNAi screening of human cells (65).

Insulin secretion is another relevant example in describing the involvement of a clock-interacting metabolic hormone, and one of the earlier evidence was observed in 3-dimentional culture of hepatocytes where insulin appeared to play a role in synchronizing the hepatic clocks (66). For many components involved in different stages of insulin action, including insulin receptor on erythrocytes (67), circadian profiles are observed. The promoter of PGC-1, an activator of gluconeogenic transcription along with FOXO1 and cortisol-receptor complex, includes insulin response sequences (IRSs) (68). Bmal1 is post-transcriptionally regulated by signals from insulin (69), while also controlling proper release of insulin. Insulin promotes postprandial Akt-mediated Ser42-phosphorylation of Bmal1 to induce its dissociation from DNA, eventually leading to nuclear exclusion. Ultimately insulin activity on Bmal1 results in the suppression of Bmal1 transcriptional activity (69). Insulin is also a target of GSK3β, which is involved in robust clock regulation by phosphorylating BMAL1 (70). More recent findings suggest that insulin and IGF-1 receptor signaling induces the synthesis of PERIOD proteins, therefore signaling the cellular clocks of feeding time of the host (71).

Focusing on the secretion of insulin itself, rhythmic release of insulin from pancreatic islets has been observed in various studies. Perfused rat pancreatic islets exhibited circadian rhythmicity in insulin release with a period close to 24 h (72). In the same study, adding melatonin advanced the insulin secretion phase by 9 h while enhancing the amplitude of oscillation, demonstrating that insulin release is under the regulation circadian clock components. In healthy humans, glucose clamping experiments showed that glucose-stimulated insulin secretion rate varies depending on the time of the day, with a nadir between midnight and 6 a.m. and a peak between noon and 6 p.m. (73). The observations from this study suggest that increase in insulin secretion during the day and decrease in secretion during the night may contribute to higher glucose tolerance and insulin response in the morning than at night. A question remains as to if the duration of prior fast for different meals throughout the day is a contributing factor in changing insulin response, as fasting duration prior to breakfast is the longest among the three meals. This was ruled out as a reason for differential insulin secretion in a study that fed subjects identical meals at 6- or 12-h intervals (74).

At a molecular level, functional clock genes are required for proper pancreatic islet function and insulin secretion. Mutations of *Clock* and *Bmal1* in mice resulted in hypoinsulemia and diabetes (75). The mutant mice had defects in size and proliferation of pancreatic islets which worsened with age, suggesting that chronic circadian disruption may affect human insulin secretion at the level of β-cells. Genes that were involved in growth, survival, and synaptic vesicle assembly were altered at the transcriptome level, leading to β-cell dysfunction. Knockout of *Clock* in human pancreatic islet cells significantly decreases both acute and chronic glucose-stimulated insulin secretion (76). Furthermore, the rhythm of insulin secretion became asynchronous with genetic clock disruption. The same study revealed that clock genes regulate the level of insulin by controlling insulin secretion rather than production. Among ~300 altered genes, regulators of insulin secretion (*gnaq, atp1a1, atp5g2, kcnj11*) as well as transcripts required for granule maturation and release (*vamp1, stx6, slc30a8*) were affected. To test whether an independent pancreatic clock regulates the secretion of insulin, both global and β-cell specific *Bmal1* deletion were tested in a study (77), and both resulted in insufficient glucose-stimulated insulin secretion, leading to β-dysfunction and diabetes. *Bmal1*-knockout mice lose the rhythms in insulin and are locked into the trough stage of insulin secretion (78), and experience increased risk of obesity under high-fat diet. Although the exact mechanism of clock control in insulin release is still under investigation, CLOCK/BMAL1 heterocomplex is thought modulate rhythmic metabolic activities in pancreas together with cell-type specific enhancer PDX1 (79) in a distinct manner. Another study has found that the presence of pineal gland is required for proper synchronization of metabolic rhythms including glucose-stimulated insulin secretion (80). Indeed, when mRNA expression levels were compared between cultured human islets from deceased donors, *Per2, Per3*, and *Cry2* were under-expressed in type 2 diabetes mellitus (T2DM) patients compared to healthy donors (81), alluding that metabolic function correlates with robust clock rhythms. The donors in this study included both men and women of varying ages for both healthy and T2DM groups.

As the above examples illustrate, robust, and synchronized circadian rhythms in the periphery are required for metabolic health of mammals. Disruption of the rhythms induced by mutation of clock genes or restriction of food is detrimental for metabolic function. Restricting food access in a manner antiphase to the animals' natural sleep/wake cycle effectively resets the phase of the peripheral clock oscillations, uncoupling from the central pacemaker in the SCN (82). Thus, food availability acts as the primary *zeitgeber* for the peripheral clocks in metabolic organs such as kidney and liver, while leaving the SCN to be coupled to the light/dark cycle. The glucocorticoid signaling from the SCN acts as an inhibitor in feeding-induced decoupling, resulting in slower phase resetting for feeding time changing from active to rest period. On the other hand, a recent study exploring methods for mitigating jet-lag illustrates the advantage of synchronized light and feeding cues. In mice, changing the feeding cycle in synchrony with the light/dark cycle results in faster re-entrainment of circadian rhythms, due to the extra-SCN oscillators phase-resetting to the feeding cues immediately and synchronizing with the SCN clocks right away (83). The rate at which peripheral clocks re-entrain to the feeding rhythms and decouple from the SCN are different for each organ, with liver resetting the more rapidly than kidney, heart, or pancreas (84). However, all organs completely re-entrain in phase to the feeding rhythms after 1 week of restricted feeding. However, the resulting rhythms desynchronized from the SCN are less robust, having oscillations with smaller amplitude. The disrupted synchrony between central and peripheral clocks is quite common due to lifestyle choices and occupational demands of the modern society. The next section will examine specific human behaviors that cause metabolic implications in relation to disruption in circadian rhythms.

## Metabolic Syndrome and Behavioral Circadian Disruption

**Figure 2:**
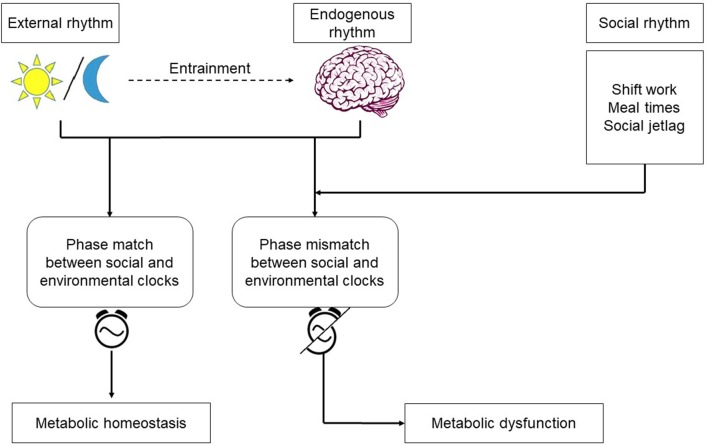
Endogenous rhythms and environmental cues: the central pacemaker in the brain is entrained by the light/dark cycle. Shift work, irregular meals, and social jetlag are behaviors which cause misalignment between the environmental and social clocks. Phase mismatch between the clocks increases the risk for metabolic dysfunction.

The metabolic syndrome is a cluster of metabolic abnormalities, including obesity, dyslipidemia, hyperglycemia, and hypertension increasing the risk for heart disease, stroke, and diabetes (85). The spectrum of disorders representing metabolic syndrome continues to increase in the industrialized world. With an incidence rate estimated to be between 25 and 40% (ages 25–64) the syndrome is becoming a major public health issue. Although genetic and environmental factors, such as increased food intake and lack of physical activity, have been known to contribute, it is becoming increasingly more clear that behaviorally-driven disruptions in circadian rhythms –a light at night or work during the rest period—substantially contribute to the disease (86). The central clock in the SCN plays a major role in maintaining metabolic homeostasis by regulating the systemic metabolic rhythmicity (87). SCN control of blood glucose level illustrates the importance of the central clock's role in modulating the metabolic activity to the environmental cues. During the active phase, glucose is taken up by liver and muscle cells via insulin dependent transporters GLUT4 and GLUT2, which are partly regulated by the clock (88). During the inactive phase, depleted glucose is replenished by glucose excretion from the liver by GLUT2 (89). As summarized in Figure 2, circadian disruption resulting from altered behaviors that create a mismatch between the host's endogenous rhythms and environmental cues, often manifests itself in the form of modifications to the oscillatory characteristics of the circadian rhythms in peripheral tissues and organs, including: reduction in the amplitude of oscillations, loss of synchrony, and peripheral clock phase misalignment relative to the SCN. Given the established circadian regulation of metabolic process (90–92), it is only natural to expect that behavioral and environmental factors leading to circadian dysregulation would adversely impact metabolism.

## Temporal Eating Patterns and Time-Restricted Feeding

Time restricted feeding studies with animals have tried to deepen the understanding about the effects of circadian misaligned meals on hosts' metabolism (93, 94) while few studies have considered implications in humans (95). Consistent with the observation in shift workers, mice fed with high-fat diet during the light (inactive) phase gained significantly more weight than mice on high-fat diet during the dark (active) phase (96). The increased risk of weight gain in day-time fed mice is accompanied by misalignment in peripheral clocks with the SCN (96). In contrast, restricting feeding to the night time (active phase for mice) leads to robust oscillation of peripheral rhythms and prevent obesity (52), although there is no difference in between caloric intake for restricted feeding group and *ad libitum* group. Interestingly, mice on high-fat diet under *ad libitum* schedule become obese, but mice consuming the same diet under restricted feeding during the dark phase consume reduced amount of food and are protected from obesity (97). Mice on restricted feeding with high fat chow had food available to them for 4 h in the middle of the active period, and their overall daily caloric intake was similar to the group of mice that had low fat food available *ad libitum*. However, these mice still showed decrease in body weight, reduced cholesterol levels, decreased in TNF-α levels, and improved insulin sensitivity, highlighting the health benefits of robust peripheral clock oscillations. Recent evidence suggests that the benefits of clock oscillations are mainly due to sustaining daily rhythms and balance between nutrients and cellular stress responses. As restricted feeding prevents metabolic syndrome in the absence of circadian clocks, rhythmicity of clock outputs may give more health benefits than the clock itself from the metabolic standpoint (98). The relationship between diet composition and circadian rhythms appears to be bidirectional since high-fat diets have also been shown to adversely impact circadian rhythms (99).

The increased metabolic risk associated with shift work may be related to the altered eating patterns due to work schedules, at least in part (100). Shift work can lead to irregular meal patterns due to both biological and social mismatch. Night workers experience a decrease in appetite because the hypothalamus that controls the eating behavior is central to synchronizing the clocks of the body to the environmental light/dark cycle. Furthermore, shift work forces the workers to eat at times different from their families and friends, creating a difference in both the timing of food intake and nutritional composition of the meals (100).

Irregular feeding patterns drive disturbances in homeostatic control through endocrine signaling (101) as evidenced by altered level of satiety hormones such as ghrelin and leptin upon sleep deprivation (102). Reduced leptin level is observed with mismatched phase relations between behavioral pattern and circadian rhythms, independent of the lack of sleep (103).

The majority of studies tend to confirm that the total caloric intake between day workers and shift workers is comparable, despite differences in the distribution of the meals during a 24-h day between the two groups (104, 105). The meals tend to become more irregular during shift work, taking snacks rich in carbohydrate and fat, as the meal times are driven more by work schedule rather than hunger (106). Furthermore, the quality of the meals does not match the day meals, possibly due to ease of preparation or the limited availability of food at workplace (100). However, such practical constraints may not be the only reason for difference in meal composition as a study conducted with nurses working night shifts found that workers opted for cold snacks when hot prepared meals were available (106). Irregularity of meals resulting from shift work could contribute to increased risk of metabolic syndrome in some part, as evidenced by population-based cross-sectional studies indicating that middle-aged men and women were more prone to metabolic syndrome if they were irregular as opposed to regular eaters (107). Furthermore, night eating syndrome (NES), where more than 20% of the daily caloric intake occurs after the evening meal, is positively associated with BMI and binge eating. It also occurs more in males compared to females (108). Interestingly, increasing meal frequency is positively correlated with lower risk of obesity, whereas skipping breakfast increases the risk of obesity (109). People who concentrated their caloric consumption earlier in the day compared to later in the day were more likely to lose weight (110).

## Shift Work

Epidemiological studies seem to point to a strong link between shift work and developing metabolic syndrome (111) likely through a complex interplay between circadian rhythms and sleep disruption (112). This is a growing concern as shift work is an unavoidable part of the modern society. According to 2004 data from Bureau of Labor Statistics, ~15 million Americans work full time on night shifts, evening shifts, or otherwise irregular shifts arranged by their employers (113). An extensive number of studies suggests that chronic disruption of circadian rhythms due to shift work, especially rotating shift work, results in negative health effects, including metabolic disorders, cardiovascular disease, and cancer (114). However, a clear mechanism that links circadian disruption to the development of metabolic syndrome is yet to be established. Longitudinal studies have demonstrated that alternating shift work was associated with increase in BMI over a 14 year period (115) and have established shift work to be a risk factor for weight gain (116). By and large, individuals who engaged in night shift work for longer period of time were at a greater risk for obesity compared to workers who started as day workers and switched to night shift, suggesting that chronic exposure to a life style mismatching the internal clock to environmental cues leads to higher risk for weight gain. An observational study conducted on nurses' cohort also found that exposure to night work can lead to more frequent weight gain (117).

Population studies indicated that obesity and increased triglycerides were more prevalent in shift workers for women and some age groups in men, even after adjusting for age, and socioeconomic factors (16). Hypertriglyceridemia is frequently observed in patients suffering from obesity, diabetes, and stroke, often accompanied by low level of high-density lipoprotein (HDL) cholesterol (118, 119). However, it is difficult to determine if the increased risk of metabolic syndrome is due to the circadian disruption or the other factors involved in shift work. Indeed, comparing shift and day workers performing similar duties, more job strain, and higher total and at-work physical activity were observed for shift workers compared to the day workers (120). After accounting for potential covariates such as physical activity and job strain, the study determined that shift work is still highly associated with metabolic syndrome. Furthermore, shift workers exhibit different meal distribution patterns from day workers, as expected, and further analysis indicated that while caloric intake during breakfast and lunch is usually favorable for good health, high energy intake at lunch is deleterious to shift workers (120). The effect of meal distribution will be discussed in more detail in a later section.

In addition to factors contributing to metabolic syndrome such as obesity and high triglycerides, shift work is also correlated to abnormal glucose levels, likely indicating that alternating shift work increases the risk of impaired glucose metabolism (121). In both young and middle-aged women, a positive association was found between T2DM and accumulation of years in rotating night-shift work, although the relationship was not strong if body weight is taken into account (122). Short-duration and poor quality of sleep increases the risk of T2DM, marked by an increase in hemoglobin A1c (123).

Efforts aiming at understanding the implications of eating and sleeping at different phases of the circadian demonstrated that such abnormalities resulted in not only major phase shifts of the cortisol rhythms, but also decreased leptin and increased insulin levels, increased mean arterial pressure but, in some cases, resulted in glucose response typical of prediabetic state (103). Therefore, circadian misalignment resulting from shift work is accompanied by adverse cardiometabolic implications.

Overall, chronic exposure, rather than acute exposure, to circadian disruption seems to increase the risk for metabolic syndrome. In a prospective study conducted among Belgian men, the risk for metabolic syndrome gradually increased with accumulated years of rotating shift work (124). In addition to metabolic biomarkers such as body mass index, waist-hip ratio, and fasting insulin level, leukocyte count was also correlated with shift work (125). Since leukocyte count is a biological marker for systemic inflammation, its association with metabolic syndrome and cardiovascular events should be further explored. Given the well-established links between low-grade, chronic inflammation, and obesity (126), it is very interesting to identify the positive correlation between shift work and systemic inflammation biomarkers (127).

## Chronotype and Social jetlag

There is a large variability in the way humans organize their sleep and wakefulness during the 24-h day. The manifestation of a person's underlying circadian rhythms is known as their chronotype (128). The chronotype is usually assessed by questionnaires including questions about sleep habits and shows a normal distribution ranging from very early chronotype to very late chronotype (128–131). Falling asleep and waking up at different times is not the only varying factor in human sleeping behavior. People also sleep for different durations, which adds ambiguity to chronotype determination. Sleep time and sleep duration appears to be two independent traits, as these separate when factor analysis is performed (129). However, there is an association between chronotype and duration of sleep on work and free days. People with late chronotypes exhibit the largest difference in sleeping time between work days and free days. This group of people wake up earlier than their biological wake up time during the work days due to work schedule, resulting in social jet-lag. Social jetlag is defined as the difference between the social clock and biological clock (128), usually due to work and social constraints (132). Since the circadian clock determines when people can fall asleep, people with later chronotypes often accrue sleep debt during the work week to be compensated on free days, which may cause conflicting schedules with family members and friends, leading to social/psychological stress that may worsen sleep disorder. Mutations in *per2, cry2*, and *csnk1d* have been shown to cause advanced sleep phase disorder, while a mutation in the *cry1* gene results in delayed sleep phase disorder. All of these are negative feedback protein-encoding genes. Genome-wide association studies of chronotype have both confirmed associations between specific genetic mutations and chronotype (133).

In addition to genetic (134, 135) and environmental factors (130), chronotype is also dependent on age. Chronotype is usually early in childhood, then is delayed as people mature, reaching the latest stage at around age 20 (129). From then on, early chronotype gradually returns as humans age. Women matures in chronotype a little bit earlier than men until age 30. However, the difference in chronotype between the sexes disappears around 30, and men become more morning-oriented than women starting at 45 years of age (136). Since this is the average age for menopause, the change in chronotype over a life span may be at least partly due to endocrine signaling (137). Given that different chronotypes (early vs. late) characterize diverse phase relations between endogenous rhythms and *zeitgebers* it was recently established that metabolic disorders are highly correlated with chronotype. In fact, late (evening) chronotypes are associated with diabetes and metabolic syndrome (138).

Social jetlag positively correlates with increased risk of adverse endocrine, behavioral, and cardiovascular risk profiles in apparently healthy subjects, putting them at a risk of developing metabolic diseases (139, 140). It has been estimated that the difference between the social clock and biological clock is >1 h in 70% of the individuals and >2 h in 1/3 of the population. The metabolic and mental health implications of mismatching clocks should be carefully considered in discussions of Daylight Savings Time as well as work and school start times (141). Social jetlag, along with short duration of sleep, appears to be a predictor of BMI, especially in over-weight individuals (141).

Often social jetlag is further accompanied with higher risks of diabetes and inflammation (142). Among non-shift workers, social jetlag was associated with numerous clinically assessed measurement indicative of metabolic diseases and obesity. Within the obese group, higher social jetlag levels were observed for metabolically unhealthy individuals, defined with biomarkers such as high waist circumference, high blood pressure, low HDL cholesterol, high glycated hemoglobin (risk factor for diabetes), and high triglycerides. In the metabolically unhealthy obese group, social jetlag was also correlated with elevated glycated hemoglobin and high-sensitivity assays of C-reactive protein (hsCRP), an indicator of inflammation.

Social jetlag is also associated with additional indicators of cardiometabolic risks, including lower HDL cholesterol level, higher triglycerides, higher fasting plasma insulin, insulin resistance, and adiposity, even after taking multiple covariates into account (143–145). Evening chronotypes associate with low level of HDL cholesterol, while social jetlag positively correlates with lower HDL cholesterol, higher triglycerides, higher fasting plasma insulin, insulin resistance, and adiposity (143). In this study, cofounding factors such as subjective sleep quality, depression, and health behaviors were adjusted for. The results of these studies demonstrate that living against the internal clock is contributing to the epidemic of obesity in industrialized societies, and improving the synchrony between the social and biological clocks may be one of the approaches for fighting obesity (141).

## Diet and Microbiome

The gut microbiota, the mixture of microorganisms inhabiting living the intestine, has been demonstrated to play a critical role in insulin resistance, obesity, and metabolic syndrome (146). Furthermore, several studies have proposed that aside from the well-studied circadian *zeitgebers* such as the light/dark cycle and meal timing, gut microbiome may influence the rhythmic expression of the host's internal body clock (147), while at the same time composition oscillations of the gut microbiome during the day further strengthen the bidirectional interaction between the gut microbiome circadian rhythms and the host's circadian rhythms (148, 149). Therefore, exerting an influence directly in the gut and through modifying the circadian rhythms.

The regulation of circadian rhythms and gut microbiota appears to be reciprocal. In germ-free mice fed either low or high-fat diets, impaired central and hepatic clock gene expression is observed even when the light/dark signals persist (150). This observation leads us to believe that the existence of microbiota is necessary for proper functioning of circadian clocks in the host. The host's internal clocks receive diurnal variation in microbial signals in 2 folds. The microbiota itself exhibits rhythmic community composition, metabolite secretion, localization, and adherence to the intestinal epithelium (150, 151). Therefore, the gut epithelium experiences differential bacterial species and their metabolites depending on the time of the day. In addition, toll-like receptors (TLRs) in intestinal epithelial cells that detect microbial metabolites are rhythmically expressed (152), adding another layer to the circadian rhythms of microbial signals that peripheral organs are exposed to.

**Figure 3:**
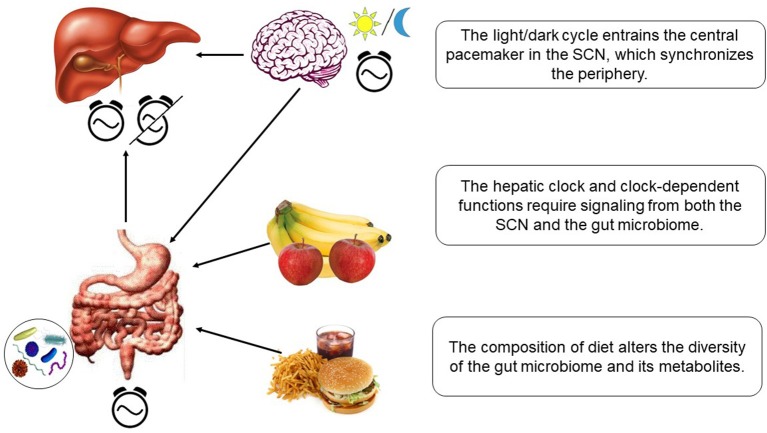
Hepatic clock and metabolism is regulated by diet-dependent gut microbiome and light-dependent SCN.

The circadian microbiota signal, particularly short chain fatty acids such as butyrate, is known to reset the hepatic circadian gene expression in addition to the intestinal clock gene expression (150, 151). Transcriptomic analysis of the liver in germ free (GF) mice and specific pathogen-free (SPF) mice shed some light onto the microbiome-liver axis (153). Axenic mice exhibit reduced amplitude and phase delay in core clock genes and altered liver transcriptome. The gut microbiome and its metabolites seem to play an essential role in activating some of the key nuclear receptors in the liver. The expression level of the nuclear receptors such as PXR, CAR, LXR, and PPARα, related to detoxification or lipid metabolism, were altered only slightly. However, the target genes of these receptors showed very little expression or dramatic dampening of the rhythms. The gut microbiome most likely influences the liver via portal vein in which the bacterial metabolites travel to the liver. The influence of gut microbiome on hepatic clock is visualized in Figure 3.

The variation in enteral microbial structure and function is dependent on dietary composition and meal timing. High fat diet changes the community composition of gut microbiota, reducing the microbial diversity, and blunts the daily changes of luminal gut microbiota abundance (150). The diet-dependent diurnal patterns of metabolite production by the microbes directly modulate hepatic clock gene expression. For conventionally raised mice, high fat diet causes circadian disruption and exposes the animals to risk of obesity. The host's internal clock is perturbed because its liver is exposed to the changes in microbe-dependent metabolites. However, germ-free mice stay lean regardless of the composition of diet. The hypothesis is that diurnal cues from the gut microbiota is missing in these animals, resulting in dampened circadian gene expression even under the presence of light/dark signals. The disturbed rhythms then lead to a heightened metabolic state, resulting in lean body mass regardless of the dietary composition. Since the composition of the diet alters the diversity of gut microbiome while also influencing the proper function of internal rhythms in the peripheral organs, person-to-person variability in metabolism could result from the type of food one chooses to consume as well as the meal times and regularity. The gut microbiome is also under the regulation of sex hormones, which will be discussed in the next section.

In humans circadian oscillations are observed in the relative abundance of 60% of the total gut bacteria, representing ~15% of various bacterial taxa (150, 151, 154). The circadian rhythmicity of the gut microbiota composition may be due to the signals from the host's peripheral circadian rhythms. The evidence for this is that disrupting the subject's circadian rhythms by the means of jetlag or clock gene mutation abolishes the oscillations in composition of the enteral microbiota (155, 156). The circadian rhythmicity of the microbe itself may also play a role, as at least one species of gut microbiota (*Enterobacter aerogenes*) exhibit endogenous circadian rhythms in swarming and motility while being entrained to melatonin secretion (157).

## Interpersonal Variability in Metabolism: Sex, Race, and Ethnicity

In addition to the degree of misalignment in circadian rhythms due to work shift and life style choices, there are additional factors that may cause person-to-person variability in metabolism. The regulation and progression of metabolic disease differs between males and females due to the effects of gonadal hormones such as estrogens and androgens on the brain and peripheral organs (158, 159). The asymmetry in metabolic homeostasis between the sexes are due to evolutionary paradigm for females to resist the loss of energy state and males to increase muscle mass and exert energy from storage promptly (160).

The gonadal hormones are responsible for sex differences in circadian timing, which then influences metabolic homeostasis. The SCN, where the master clock for mammals reside, exhibit morphological differences between male and female mammals, in volume, number of synapses, and length of rostrocaudal axis (161). The electrical activity in the SCN is also sex-dimorphic. In the SCN core region, the action potential threshold and greater amplitude for males is higher during the dark phase (162), suggesting that females may adjust more easily to new patterns in environmental cues due to low amplitude. In the SCN, androgen and estrogen receptors are present, with males having higher level of androgen receptor expression in human (163) and females having higher level of estrogen receptor alpha expression (164). Male mice that received gonadectomy show longer circadian rhythm period, and treatment with testosterone or dihydrotestosterone can reverse these responses (161), showing the necessity of androgen for entrainment of internal rhythms to environmental cues. Sex difference also exists in HPA functioning through differential HPA response to stress and rhythms or hormone secretion (161). Proestrus female rodents show more increase of basal plasma ACTH and CRH mRNA expression under stress (165). Under basal and stress conditions, female rats show higher level of glucocorticoid secretion, along with a higher peak in its circadian rhythms (166). In females, corticosterone levels increase and decrease with the estrus cycle, as estrogen increases ACTH release by increasing the duration of secretion (167). In contrast, androgen has an inhibitory effect on the magnitude of ACTH release (166). In addition to the morphological differences and SCN signaling activities, gonadal hormones also appear to affect sleep behavior, and homeostasis. Women have a tendency to go to sleep earlier than man throughout childhood and adulthood; however, the difference disappears at menopause (129). Women also report poorer sleep quality and are more likely to develop insomnia compared to men, despite reporting longer hours of sleep (168). Sleep debt accumulates more quickly in women than men, and has a greater negative impact on women's health (169). As the peripheral clocks entrained by the HPA axis and cortisol interact closely with the metabolic activities of the host, the sex differences in the regulation of these would manifest to variability in metabolism as well. In addition to the gonadal hormones, the effects of sex chromosomes on sex differences in metabolism was recently explored (170). Gonadectomy has differential effects on energy and metabolism of gonadal males and females. Gonad-intact females consume food earlier in the day than males. Gonadectomy phase-advances the feeding time, especially in XX mice. However, gonadectomized XY mice still show earlier acrophase of feeding compared to gonad-intact females (170).

The sex hormones appear to have an influence on the microbiome of the host, as evidenced by male and female mice exhibiting differences in microbiome composition (171). Gonadectomy-associated changes in gut microbiota and bile acid composition can be prevented by administration of testosterone, further confirming that sex hormones, and related clock genes are involved in controlling the enteral microbiome (171). The difference in gut microbiome of rats is at the single strain level, meaning that the sex differences could be masked by individual genetic variation and dietary changes (171). In humans, the sex differences in gut microbiota are observed at the bacterial phylum, genus, and species levels (172), showing more distinction between the microbiota of two sexes compared to rodents. The differences are also associated with obesity or BMI (172). For example, the abundance of *Bacteroides* genus decreases with BMI in men, but remains unchanged with increasing BMI in women, resulting in lower abundance in men than women at high BMI (172). The discrepancy between gut microbiota of men and women appears to have an influence in the gender differences in the susceptibility to cardiometabolic diseases. For example, females are more susceptible to type 1 diabetes (T1D) in both humans and rodents. However, the difference does not exist for germ free mouse model of T1D (173). The gut microbiome of the T1D mouse model is similar in composition for both sexes up to puberty, but in adulthood, males have greater abundance of some genera, including *Roseburia, Coprococcus*, and *Bilophilia* (173, 174). Furthermore, castration of male mice leads to a gut microbiota composition similar to that of the females and increases the T1D incidence (173, 175).

Sex, race, and ethnicity play intertwined roles in the development of metabolic syndrome and related comorbidities (176, 177). Recent epidemiological studies reveal that although adolescent African-American males are much less likely to be diagnosed with metabolic syndrome that non-Hispanic whites, even though they are more likely to be obese. On the other hand, non-Hispanic African-American women are more likely than non-Hispanic white women to develop metabolic syndrome. Recent evidence also suggests that obesity in white, post-menopausal women is not associate with increased cardiovascular risk, unless accompanied by metabolic syndrome, whereas overweight, or obese, African-American women had elevated cardiovascular risk, even if they did not have metabolic syndrome (178).

Given the intimate connection between metabolic syndrome and circadian rhythms, it is interesting to explore what is broadly known regarding the relationships between race/ethnicity and circadian rhythms to rationalize some of the aforementioned observations. Recent studies have compared the endogenous circadian rhythms between African-Americans and non-Hispanic European Americans (179) to determine that the former had slightly longer period than the latter, likely the result of adopting more favorable periods prior to migrating out of areas near the equator. Even more interestingly, males of European American ancestry had longer period than females. A hypothesis for this observation is that out of the equator area, longer circadian period is advantageous in tracking dawn, which is important for males as hunters but not so much for women as gatherers (179). The evolutionarily driven adaptation of the endogenous (free running) rhythms could possibly provide clues as to the differential response of various ancestry characteristics on circadian misalignment. This was test in a study assessing the impact of chronodisruption in African-Americans and non-Hispanic European-Americans (180). Interestingly the longer period of European-Americans was associated with longer phase delays following chrono disruption. The key message being that the endogenous rhythms, as evolutionary developed, determine the ability of subject of different decent to adapt to circadian misalignment. These finding could potentially shed light on the race dependencies of chrono-disruption.

However, deciphering individualized effects of circadian misalignment is a complex issue. Two insightful early studies attempted to characterize the intrinsic qualities of individuals not being affected by shift work (181, 182). Interestingly, these early studies seem to indicate that individual tolerant to shift work appear to (i) exhibit more robust circadian amplitude; and (ii) appear to adjust slowly to changing light-dark schedules. It is therefore evident that genotypic differences, establishing the dynamic of endogenous rhythms, underlie the ability of the host to cope with *zeitgeber* changes. The issue of the emergence of such trade-offs in the context of circadian misalignment was further discussed in (183).

## The Circadian Clock as a Target in Metabolic Disorders

The wealth of information connecting circadian disruption and metabolic syndrome has prompted an interest in the development of pharmacological clock-targeting compounds aiming at attenuating disease symptoms by resetting dysregulated circadian rhythms (184) whereas a wide range of small molecules are currently explored as likely pharmacologic leads (185, 186). Preliminary studies have established that diets rich in certain nutritional supplements, such as selenium (187), or certain flavonoids (188) have been shown to enhance circadian rhythms with positive health implications (189). Pharmacologic studies using *nobiletin*, a flavonoid, natural polyphenol, isolated from citrus peel, demonstrated that strengthening circadian amplitude as a plausible pharmacological intervention for metabolic disorders (188). Furthermore, nobiletin was shown to ameliorate lipid metabolism in hepatocytes, mediated via modulations of the clock gene *Bmal1* (190). Similarly, nobiletin was shown to restore circadian rhythms in embryonic fibroblasts (191).

## Mathematical Modeling of Circadian Metabolism

Since the understanding of the intricate network consisting of circadian clocks and metabolic activities is still under development, mathematical modeling of the system is useful in testing hypotheses on the relationship between circadian disruption and metabolic dysfunction. In addition to aiding in understanding of interaction between metabolism and circadian rhythms, modeling can also help define optimized feeding regimen for various photic environment for healthy and diseased hosts. Over the past decade the circadian system has been modeled using different approaches such as differential equations (192) automata, and Petri Nets (193). The earliest circadian model was based on a simple feedback loop comprising of an activator and inhibitor (194). Later, the coupling of ultradian oscillators to generate a circadian rhythmicity was also demonstrated in a mathematical model (195). More recent models target the proteins that form the core clock functions in the SCN and in the periphery, such as PER, CRY, BMAL1, and CLOCK (192, 196). The models describing the core clock machinery were later utilized to study the entrainment mechanism or to describe downstream biological functions that are closely regulated to circadian clocks. For example, a mathematical model that described the entrainment of PCGs by cortisol (197) was expanded to describe the entrainment of clock genes by the light/dark cycle (198). The model was used to explore the effects of seasonal variations in light duration on the HPA axis response (199, 200). The effects of sex differences and interindividual variability on the activity of HPA axis and cortisol secretion were also modeled (201), which would have implications for the clock genes entrained to cortisol and the biological functions tied to the clock rhythms.

More recently, there have been efforts to integrate the newly discovered transcriptional/translational regulation of core circadian clock by the feeding induced signals. The integration of feeding signals transmitted through SIRT1, PARP1, and HSF1 to the clocks with a Petri Net model was studied in (202) in order to study the effects of different feeding regimens to demonstrate that circadian system can be entrained to the differential feeding rhythms and that the feeding signals were transmitted to the PCGs. The model shows that 2 or 3 meal/day feeding regimens are beneficial for robust circadian rhythms while less or more meals/day have negative effects. The integrated role of feeding/fasting cues to the hepatic circadian clock was studied by incorporating SIRT1 and AMPK as the metabolic sensors (203). The content of the diet (normal, fasting, and high-fat) were simulated as the activation pattern of AMPK, and the model predicted the observed decrease in clock gene expression for high-fat diet.

Several studies have explored the effects of circadian disruption on clock gene expression and metabolic syndrome. Along those lines, a model that takes in the feeding/fasting rhythms through NAD^+^ mediated SIRT1 activation and light/dark cycle through the HPA axis modeled with Goodwin oscillator predicted asymmetrical entrainment of clock genes to the two environmental cues was recently discussed in (204). The model was further developed to study the effect of *zeitgeber* phase mismatch on expression of gluconeogenic genes such as *G6pc* and *Pck1* (205). This model reproduced the finding that genetic circadian disruption through *Clock* gene knockout results in altered level of gluconeogenic gene expression and predicted that certain phase relations between the two *zeitgebers* can recover the wild-type expression level in knockout cells. The effect of light-feeding mismatch was also modeled for pancreatic β cells (206). Consistent with the previous findings, restricting food access to rest phase results in phase shift of clock gene expression as well as metabolic abnormalities such as hypoinsulinemia and hyperglycemia. Recent studies further help elucidated the complex regulatory impact of SIRT1 on the regulation of clock function through actions on PER2, and PGC1α and CLOCK/BMAL1 (207).

## Concluding Remarks

Organisms have evolutionarily adapted their circadian machinery to allow flexibility in processing the changing environment such as the light signal, temperature, and food availability throughout the 24-h day. However, the biological clock is greatly impacted by the modern society and technologies that imposes a lifestyle which often misaligns the endogenous rhythms from the earth's rotation. Disrupted rhythms manifest in the form of phase mismatch and muted amplitude in expression of clock genes with serious health implications including metabolic syndrome. Genetic modifications in animal models have shown that proper functioning of clock genes at a molecular level is necessary for homeostatic metabolic activity. For instance, homozygous mutation of *Clock* in mice result in suppressed metabolic rate rhythm and overall decrease in metabolic expenditure, increased risk of obesity, hyperleptinemia, hyperlipidemia, hepatic steatosis, hyperglycemia, and hypoinsulemia (208). Although mechanisms discovered in clock knockout animal models cannot directly translate to human circadian disruption, it can start to provide insight into and generate hypotheses around human physiology relating to circadian driven metabolic dysfunction. Recent findings on signal transduction of feed/fast cycles that influence the clock proteins are beginning to shed light on the mechanism of metabolic syndrome resulting from circadian disruption, but much work remains to gain comprehensive enough understanding to begin prevention and treatment of the metabolic conditions.

Shift work, variable eating patterns, and social jetlag strongly suggest that chronic exposure to misaligned rhythms is more detrimental to health compared to acute exposure. Although mathematical modeling of circadian metabolism is beginning to provide insight into effects of *zeitgeber* misalignment and implications of restricted feeding, they have not yet been able to address the question of chronic phase mismatch intensifying the degree of metabolic dysfunction. Understanding the network of clocks and metabolic components is challenging because in addition to the underlying complexity of the system, there are also many sources for interindividual variability. Inconsistencies could arise from behaviors, life style choices, genetic makeup, and socioeconomic factors, accumulating convolution in studying long-term effects. However, current understanding suggests that behavioral changes (i.e., restricted feeding) can reverse chronodisruption (205, 209) due to genetic or behavioral causes, and broadening the understanding of mechanism behind circadian metabolism would be beneficial for chronotherapy and discussion in more biologically pertinent social clock.

## Author Contributions

S-AB: wrote the manuscript. MF, VR, and HZ: edited the manuscript. IA: conceived the idea and edited the manuscript.

### Conflict of Interest Statement

The authors declare that the research was conducted in the absence of any commercial or financial relationships that could be construed as a potential conflict of interest.
